# Macroautophagy is repressed during mitosis – seeing is believing

**DOI:** 10.1080/15548627.2020.1725405

**Published:** 2020-02-20

**Authors:** Richard I. Odle, Simon J. Cook

**Affiliations:** Signalling Laboratory, The Babraham Institute, Cambridge, UK

**Keywords:** Autophagy, ATG13 (autophagy related 13), CDK1 (cyclin dependent kinase 1), mitosis, MTOR (mechanistic target of rapamycin kinase), MTORC1 (MTOR complex 1), omegasome, RPTOR/RAPTOR (regulatory associated protein of MTOR complex 1), ULK1 (unc-51 like autophagy activating kinase 1)

## Abstract

For the last two decades there has been wide ranging debate about the status of macroautophagy during mitosis. Because metazoan cells undergo an “open” mitosis in which the nuclear envelope breaks down, it has been proposed that macroautophagy must be inhibited to maintain genome integrity. While many studies have agreed that the number of autophagosomes is greatly reduced in cells undergoing mitosis, there has been no consensus on whether this reflects decreased autophagosome synthesis or increased autophagosome degradation. Reviewing the literature we were concerned that many studies relied too heavily on autophagy assays that were simply not appropriate for a relatively brief event such as mitosis. Using highly dynamic omegasome markers we have recently shown unequivocally that autophagosome synthesis is repressed at the onset of mitosis and is restored once cell division is complete. This is accomplished by CDK1, the master regulator of mitosis, taking over the function of MTORC1, to ensure autophagy is repressed during mitosis.

For better or worse, LC3B lipidation combined with lysosomal inhibitors is widely used as a “gold-standard” assay for autophagy. However, several groups have identified instances when it is inappropriate to use such assays; for example, lysosomal inhibitors such as ammonia and chloroquine actually promote LC3 lipidation by activating non-canonical autophagy. Furthermore, LC3 puncta (another common marker) can persist for approximately 30 min, making it difficult to deduce whether LC3 puncta observed during the early phases of mitosis originate from autophagosome synthesis during the preceding interphase or during mitosis itself. As an alternative we elected to study autophagy by visualizing ATG13 and WIPI2 puncta. These are early, highly dynamic structures; for example, ATG13 localizes to the developing omegasome but leaves it prior to autophagosomal budding, thus persisting for only ~3 min and enabling a far more accurate dissection of rates of autophagosome biogenesis between interphase and mitotic cells. In this way, using both fixed and live-cell imaging techniques, we were able to demonstrate that autophagy initiation is clearly repressed in mitosis, including in cells undergoing a normal, unperturbed mitosis [1]. The use of appropriate, dynamic markers of autophagy were critical in these studies and we would encourage colleagues to avoid the use of LC3 as a readout of autophagy during mitosis, unless the aim of the research question specifically demands it.

Curiously, we observed that autophagy is repressed during mitosis even when cells are deprived of amino acids or treated with the ATP-competitive MTOR inhibitor AZD8055. Indeed, phosphorylation of RPS6KB/S6K at the known MTORC1 target site, threonine 389, is reduced in mitotic cells, in line with observations made by independent groups. To understand why MTORC1 is seemingly unimportant in the regulation of autophagy during mitosis we again employed imaging. MTORC1 is recruited to lysosomes where it is activated, enabling it to phosphorylate its substrates such as ULK1, RPS6KB and EIF4EBP1/4E-BP1. By imaging endogenous GFP-RPTOR we demonstrated that MTORC1 fails to localize to lysosomes during mitosis, providing a likely mechanism for MTORC1 inactivation during mitosis. CDK1 is the master regulator of mitosis and we showed that RPTOR undergoes CDK1-dependent mitotic phosphorylation, in line with previous findings; more importantly, phosphomimetic mutation of known CDK1-dependent phosphorylation sites on RPTOR impairs the RPTOR-RRAGA interaction, suggesting that mitotic phosphorylation of RPTOR mediates MTORC1 inactivation. The continued localization of TFEB to lysosomes during mitosis suggests that RRAG function remains intact.

Because MTORC1 is inactive during mitosis, it might be expected that its known substrates involved in autophagy regulation (ATG13, ULK1, ATG14 and TFEB) would not be phosphorylated. However, this would not be consistent with the repression of autophagy. Indeed, we observed that all of these proteins are hyperphosphorylated during mitosis, including at known repressive sites within these proteins that are usually phosphorylated by MTORC1. These sites are phosphorylated in mitosis even in the presence of MTOR inhibitors (AZD8055, Torin1, PP242) arguing that MTORC1 cannot be the relevant regulatory kinase during mitosis. Instead, we suggest that CCNB1-CDK1 represses autophagy during mitosis because: (i) these sites are proline-directed serine/threonine sites, satisfying the minimum consensus motif of CDK1; (ii) their phosphorylation in cells is reversed by CDK1 inhibitors and (iii) CCNB1-CDK1 can phosphorylate fragments of these autophagy regulators *in vitro* at these sites.

Finally, we wanted to define the functional consequence of these phosphorylation events. However, it was not possible to investigate this by mutation of the relevant phosphorylation sites because this would undermine normal regulation of autophagy by MTORC1 in interphase. Furthermore, MTORC1- and CDK1-dependent phosphorylation events extend across multiple autophagy proteins suggesting a system-wide shutdown of autophagy, which is unlikely to be rescued by mutation of a single protein. Instead we assessed known functional outcomes of the repressive phosphorylation of these proteins. TFEB is exported from the nucleus just prior to nuclear envelope breakdown, coinciding with CDK1 activation, and ULK1 activity is dramatically reduced in mitotically-arrested cells, as measured by phospho-ATG14 (S29). Recent advances in development of antibodies for another ULK1 target substrate, phospho-ATG16L1 (S278), may allow future independent confirmation of our conclusion that ULK1 activity is inhibited in mitotic cells. In addition, we speculate that other autophagy regulators may come under the control of CCNB1-CDK1 during mitosis.

Conceptually it makes sense for CDK1 to coordinate the inhibition of MTORC1 (through RPTOR phosphorylation) and to also take over its functions by phosphorylating MTORC1 sites in autophagy regulators. This mechanism insulates autophagy from nutrient status, thereby ensuring that the genome is protected from nonselective macroautophagy throughout mitosis. Critically, we must emphasize again our rejection of LC3 puncta or LC3 processing as markers of autophagy during this dynamic cell cycle event; our unambiguous demonstration of autophagy repression during mitosis was dependent on the use of dynamic markers that were fit for the experimental purpose. Indeed, we think mitosis acts as an excellent case study for why authors should consider using omegasome markers as a direct and unambiguous readout of autophagosome biogenesis.
